# High-Power Short-Duration Lesion Index-Guided Posterior Wall Isolation beyond Pulmonary Vein Isolation for Persistent Atrial Fibrillation

**DOI:** 10.3390/jcm12165228

**Published:** 2023-08-11

**Authors:** Sergio Conti, Francesco Sabatino, Fabrizio Fortunato, Giuliano Ferrara, Antonio Cascino, Giuseppe Sgarito

**Affiliations:** 1 Department of Electrophysiology, ARNAS Civico–Di Cristina–Benfratelli, 90127 Palermo, Italy; 2 Faculty of Medicine, Postgraduate School in Cardiology, University of Palermo, 90127 Palermo, Italy

**Keywords:** persistent atrial fibrillation, atrial fibrillation ablation, pulmonary vein isolation, posterior wall isolation, lesion index, high power

## Abstract

**Background:** High-power short-duration (HPSD) radiofrequency (RF) ablation has been adopted to improve atrial fibrillation (AF) ablation. Although the role of HPSD is well-established in pulmonary vein isolation (PVI), fewer data have assessed the impact of HPSD when addressing extra-pulmonary veins (PVs) targets. Therefore, this study aims to determine the safety, effectiveness, and acute outcomes of HPSD lesion index (LSI)-guided posterior wall isolation (PWI) in addition to PVI as an initial strategy in persistent atrial fibrillation (Pe-AF). **Methods:** Consecutive patients who underwent ablation of Pe-AF in our center between August 2021 and January 2022 were retrospectively enrolled. All patients’ ablation strategy was PVI plus PWI using HPSD LSI-guided isolation. RF parameters included 50 W targeting LSI values of ≥5 on the anterior part of the PVs and anterior roofline and ≥4 for the posterior PVs aspect, bottom line, and within the posterior wall (PW). We compared the LSI values with and without acute conduction gaps after the initial first-pass PWI. Left atrial mapping was performed with the EnSite X mapping system and a high-density multipolar Grid-shaped mapping catheter. We compared the procedural characteristics using HPSD (*n* = 35) vs. a control group (*n* = 46). **Results:** Thirty-five consecutive patients were included in the study. PWI on top of PVI was achieved in all cases in the HPSD group. First-pass PVI was achieved in 93.3% of PVs (*n* = 126/135). First-pass roofline block was obtained in most patients (*n* = 31, 88.5%), while first-pass block of the bottom line was only achieved in 51.4% (*n* = 18). There were no significant differences compared to the control group; first-pass PVI was achieved in 94.9% of PVs (*n* = 169/178), first-pass roofline block in 89.1%, and bottom-line in 45.6% of patients. To achieve complete PWI with HPSD, scattered RF applications within the PW were necessary. No electrical reconnection of the PW was found after adenosine administration and the waiting period. The procedure and RF times were significantly shorter in the HPSD group compared to the control group, with values of 116.2 ± 10.9 vs. 144.5 ± 11.3 min, and 19.8 ± 3.6 vs. 26.3 ± 6.4 min, respectively, *p* < 0.001. Fluoroscopy time was comparable between both groups. No procedural complications were observed. At the 12-month follow-up, 71.4% of patients remained free from AF, with no differences between the groups. **Conclusions**: HPSD LSI-guided PWI on top of PVI seems effective and safe. Compared to a control group, HPSD is associated with similar rates of first-pass PWI and PVI but with a shorter procedural and RF time.

## 1. Introduction

Durable pulmonary vein isolation (PVI) is recognized worldwide as the mainstay of any atrial fibrillation (AF) ablation procedure. The current guidelines recommend PVI for paroxysmal atrial fibrillation (PAF) ablation [1,2]. However, in persistent AF (Pe-AF), the debate on which strategy to adopt is currently very active [3,4]. The common goal of all techniques proposed is to achieve complete and durable ablation. An enduring ablation procedure is a prerequisite to prevent future arrhythmia recurrences due to partially ablated tissue. Posterior wall isolation (PWI) is among the strategies used on top of PVI to improve ablation outcomes. There are several motivations behind the reason to include PWI when ablation of Pe-AF is planned. First, the posterior wall (PW) and pulmonary veins (PVs) share the same embryological origin. The two structures are strictly correlated, potentially favoring the creation of complex circuits [5,6]. Second, the intrinsic electrophysiologic characteristics of the atrial myocytes of the PW may contribute to the pathophysiology of AF [7]. Finally, in patients with Pe-AF, the PW is prone to atrial remodeling, comprising fibrosis and lymphomononuclear infiltration [8,9]. Moreover, so-called rotors have been previously reported in the PW of Pe-AF patients [10].

Nevertheless, PWI feasibility, safety, and effectiveness are still questionable. Literature data on PWI focus on non-homogeneous strategies to achieve PWI, mixed patients’ cohorts, single-center studies, or small sample sizes. In addition, most of the time, delivering durable lesions on the PW is a challenge. It has been shown that regular confirmation of PWI durability before starting the follow-up is essential, demonstrating that PWI during the index procedure is of crucial importance [11]. Ablation with contact force (CF)-sensing technology catheters with the guidance of multiparametric indexes, such as the Ablation Index (AI) or Lesion Size Index (LSI), have been linked to enhanced procedural safety and efficacy. LSI is a multiparametric index introduced to improve radiofrequency (RF) lesion formation. It incorporates time, power, CF, and impedance data recorded during RF ablation with CF-sensing technology catheters [12,13]. The LSI-guided high-power ablation with CF-sensing technology catheters might aid in the further improvement of safety while generating lasting transmural lesions. Although LSI has been proven to be a safe and effective tool to evaluate lesion quality and size in real-time during PVI, its role in PWI has not yet been widely assessed. Moreover, high-power (≥50 W) short-duration (HPSD) RF ablation is safe and effective when compared to traditional lower-power longer-duration ablation [14,15]. In addition, lesion width has been suggested to be increased in HPSD compared to conventional ablation techniques since a substantial fraction of the ablation time is the resistive heating phase [16]. Moreover, previous papers published analyzed data from Holter ECG monitoring to assess for recurrences. It is well known that continuous rhythm monitoring, such as insertable cardiac monitors, can be more accurate in detecting arrhythmic events.

## 2. Methods

### 2.1. Patient Population

Between August 2021 and January 2022, we prospectively recruited consecutive patients with Pe-AF who underwent PVI plus PWI using the HPSD setting and guided by LSI. All patients had Pe-AF as defined by the latest guidelines and had indication to perform catheter ablation [1,2]. Patient’s clinical characteristics were recorded from the hospital’s medical records. The local institutional review board approved the study protocol, and the study complied with the Declaration of Helsinki. All patients gave written informed consent before the procedure.

### 2.2. Ablation Procedure

**Figure 1:**
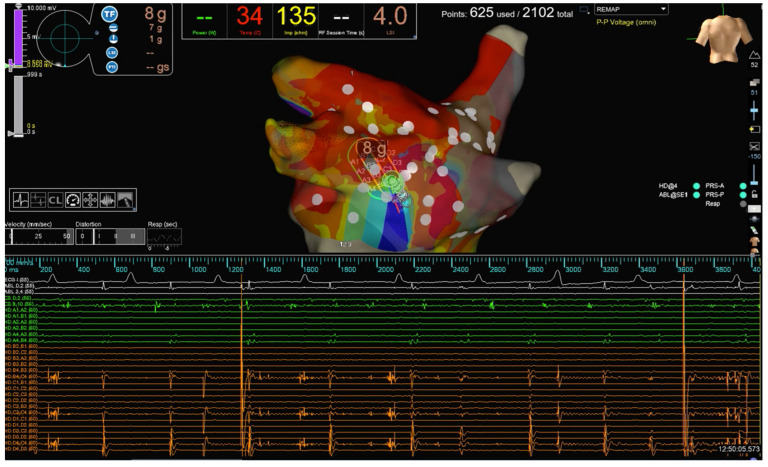
The strategy of PWI in our study. PW activity before complete isolation. The PW activity recorded on the HD Grid catheter is evident.

**Figure 2:**
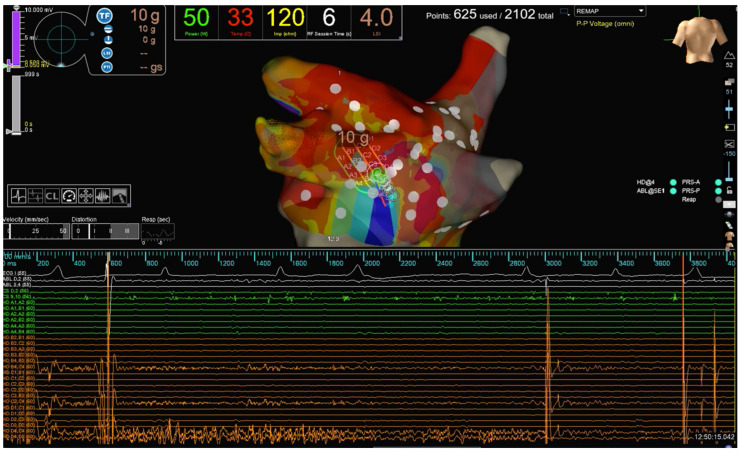
Same patient as Figure 1. HPSD (50 W) scattered RF lesions are performed to achieve PWI in one of the patients enrolled. PWI recorded by the HD Grid catheter during RF delivery is evident.

A pre-procedural transesophageal echocardiography was performed to exclude left atrial and appendage thrombosis. Antiarrhythmic drugs (AADs) were discontinued at least three half-lives before the ablation for class I, and four weeks before for amiodarone. All procedures were performed as previously described [17]. Briefly, an uninterrupted anticoagulation strategy was adopted in all cases. Intra-procedural intravenous heparin administration was given with an initial bolus of 50–100 IU/kg, followed by a continuous infusion (1000 IU/h). The activated clotting time was maintained at ≥300 s and checked every 20 min during the procedures. A 6F deflectable decapolar catheter was inserted through the right femoral vein and advanced into the coronary sinus. Transseptal access was obtained twice using a BRK XS needle and two non-deflectable sheaths (SL1 8.5F, Abbott Medical, Abbott Park, IL, USA). LA geometry and high-density bipolar LA voltage (>2000 points) were performed using the EnSite X mapping system and the Advisor HD Grid SE. A baseline bipolar LA voltage map was created in sinus rhythm before ablation. PVI was performed with RF energy point-by-point, and RF delivery was initiated at a stable CF ranging from 10 to 20 g. When ablating closely to the esophagus, our target CF was limited to 5–8 g but was never greater than 10 g. In our LSI-guided approach, RF energy delivery was terminated when a target LSI of 5.0 for the anterior aspect of the PVs and the anterior aspect of the roof and a target LSI of 4.5 for the posterior part of the PVs and PW were reached. The target inter-lesion distance was <5 mm. PWI was achieved by connecting the antrum of the PVs with an anterior cranial roof line and a caudal line at the floor level of the LA. As part of our standard protocol in Pe-AF ablation, we usually deliver additional RF lesions across the entire PW to achieve PWI (Figure 1 and Figure 2). All procedures were performed using an esophageal probe to monitor the endoluminal temperature (Esotherm Plus, Fiab, Florence, Italy). RF was stopped if the endoluminal esophageal temperature reached 38 °C, considered the cut-off limit. The acute endpoint was to achieve complete PVI and PWI, confirmed by the Advisor HD Grid SE positioned in each PV and by differential pacing maneuvers. After a waiting period of 20 min from the last ablation, PVs were checked with the Grid to assess for spontaneous PV reconnection. If PV reconnection was not documented, intravenous adenosine was given to unmask dormant conduction. CF and LSI data were recorded for PVI and PWI. RF, fluoroscopy, procedural times, and incidence of procedural and peri-procedural complications (vascular complications, cardiac tamponade, thromboembolism, atrio-esophageal fistulas, phrenic nerve palsy, pulmonary vein stenosis, etc.) were also collected. After the procedure, all patients received an implantable loop recorder (Reveal LinQ Medtronic, Minneapolis, MN, USA, or Confirm RX, Abbott Medical, Abbott Park, IL, USA). Before discharge, a transthoracic echocardiography was performed to exclude pericardial effusion.

### 2.3. Patient Follow-Up

All patients enrolled in the study performed a visit in the outpatient clinic at 3, 6, and 12 months. At each visit, a standard 12-lead ECG was recorded. Oral anticoagulants were stopped according to the CHA_2_DS_2_-VASc eight weeks after ablation. AADs were withdrawn at three months or continued at the physician’s discretion. In addition, after the 90-day blanking period, data recorded from the ILR were remotely and on-site collected to evaluate the occurrence of atrial tachycardia (AT), atrial flutter (AFL), and AF episodes. Each follow-up focused on the assessment of atrial arrhythmia-related symptoms and AF burden. Atrial arrhythmia recurrence was defined as any documented episode of atrial tachycardia (AT), atrial flutter (AFL), and AF lasting longer than 30 s. The AF burden was calculated as the percentage of time in AF between each follow-up visit based on manually adjudicated episodes. Any arrhythmia observed within three months after ablation was defined as early AF and not considered an arrhythmia recurrence. Redo was always performed after the 90-day blanking period.

### 2.4. Statistical Analysis

This was a single-center study. Patients were enrolled prospectively. All clinical characteristics are reported as descriptive statistics. Continuous variables are expressed as mean ± standard deviation. Categorical variables were reported as percentages. A *p*-value of <0.05 was considered statistically significant. Arrhythmia survival curves were created by the Kaplan–Meier method. All statistical tests were performed using SPSS for Windows 25.0 (SPSS, Chicago, IL, USA).

## 3. Results

**Figure 3:**
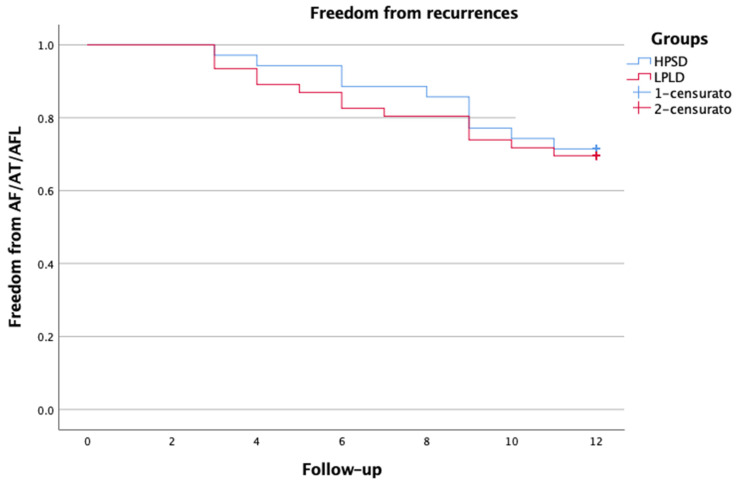
Kaplan–Meier survival analysis.

A total of 35 patients with symptomatic Pe-AF were consecutively included in the study. The baseline clinical characteristics of the patient population are reported in Table 1. All patients underwent at least one attempt of electric cardioversion before the procedure. The procedural characteristics are reported in Table 2. In all cases, PWI guided by LSI using HPSD settings was performed after PVI. First-pass PVI was achieved in 93.3% of PVs (*n* = 126/135). First-pass roofline block was obtained in most patients (*n* = 31, 88.5%), while first-pass block of the bottom line was only achieved in 51.4% (*n* = 18). When comparing the HPSD group to a control group of patients in which PWI was performed with low-power long-duration ablation (30–35 W), there were no significant differences; first-pass PVI was achieved in 94.9% of PVs (*n* = 169/178), first-pass roofline block in 89.1%, and bottom-line in 45.6% of patients (*p* = ns). Scattered RF applications—in HPSD—within the PW were delivered to achieve complete PWI. We did not observe electrical reconnection of the PW after the waiting period and adenosine administration. Procedure and RF times were significantly shorter in the HPSD group compared to the control group, with values of 116.2 ± 10.9 vs. 144.5 ± 11.3 min, and 19.8 ± 3.6 vs. 26.3 ± 6.4 min, respectively, *p* < 0.001 (Table 3). Fluoroscopy time was comparable between both groups. No procedural complications related to HPSD settings were observed. One patient had a vascular complication but did not require surgery. The mean length of hospital stay was 2 ± 1.2 days. At the 12-month follow-up, 71.4% of patients remained free from atrial arrhythmia. There were no significant differences compared to the historical control group (71.4% vs. 69.5%, *p* = ns) (Figure 3). At the 1-year follow-up, 31.5% of patients were on AADs. Post-procedural AF burden was significantly decreased from 89% to 22% (*p* < 0.0001).

## 4. Discussion

In this study, we evaluated HPSD catheter ablation with PVI plus PWI guided by LSI using the TactiCath SE ablation catheter to treat patients with Pe-AF. Unlike previous studies, we included only patients with Pe-AF in which an extensive PWI has been performed with HPSD and LSI guidance and a strict follow-up with continuous rhythm monitoring given by ILR. Compared with a group of Pe-AF patients treated with low-power long-duration ablation (35 W), we reported a shorter procedure and RF time but no differences in outcomes or complications.

HPSD ablation creates lesions with equal volumes compared to standard settings but wider and less deep lesions, most likely due to the increased resistive heating component. Previous studies evaluating catheters without CF-sensing capabilities showed that high-power RF ablation settings (50 W) resulted in better long-term freedom-from-AF with shorter fluoroscopy and procedural times without increasing the complication rates when compared to low-power (35 W) ablations [14,15,18,19,20]. The introduction of CF-sensing ablation catheters resulted in an improvement in outcomes both in paroxysmal and in Pe-AF ablation. Hussein et al. showed that using CF-sensing catheters for Pe-AF resulted in significantly higher freedom from AF at 1-year follow-up than non-CF catheters (72.4% vs. 53.6%) [21]. The TOUCH AF trial was a multi-center randomized trial investigating the effect of CF-sensing ablation catheters in Pe-AF [22]. Even with the minimalist technique adopted in the TOUCH AF trial—PVI plus roofline—the authors reported a significantly higher degree of freedom-from-arrhythmia recurrence rate than previous studies where no CF catheters were used. In contrast to previous papers, we assessed the LSI-guided 50 W PWI beyond PVI. We adopted a target LSI of 5.0 for the anterior aspect of the PVs and the anterior aspect of the roof and a target LSI of 4.5 for the posterior part of the PVs and PW. Previously, Parwani et al. demonstrated that a target LSI of 5.0 for the anterior segment of the LA wall and 4.5 at the posterior aspect of the LA wall can be safely reached using the TactiCath CF sensing catheter with an energy delivery of 50 W [23]. The LSI is a good predictor of lesion size in low-power ablation settings. An LSI of 4.5 at the posterior left atrial wall and an LSI of 5.0 at the anterior left atrial wall have already been suggested by previous studies to predict effective lesion formation [12,18,24].

Previous studies have demonstrated that PWI is a feasible strategy for catheter ablation of Pe-AF [25,26,27,28,29]. These findings were confirmed in a recent meta-analysis of multiple randomized clinical trials demonstrating the incremental benefit of PWI [30]. However, how to perform isolation of the PW remains a challenge and is still a debatable and controversial point. A successful and persistent PWI procedure is technically laborious because of the complex anatomical structure of the atrial musculature. Moreover, even if a conduction block along the lines is achieved, the occurrence of gaps over time cannot be ruled out, and thus dormant conduction may take place during the follow-up. In their paper, Tamborero et al. stated that PWI created with linear lesions does not improve the clinical outcome of PVI [31]. This is because nearly 70% of patients had reconnection of the roof line or recurrence of electrical activity within the PW that led to AF and AFL. Sayuri et al. showed a reconnection of PW in 65% of patients after the second procedure [32]. In addition to CF sensing information, more objective indexes, such as AI or LSI, have been developed to improve the quality of RF lesions and have been advocated to standardize the outcomes of AF ablation, reducing the inter-operator differences. Recently, the CAPLA randomized clinical trial assessed the role of empirical PWI in patients with Pe-AF [33]. The trial did not show additional advantages in the group of patients randomized to PWI and raised doubts about this strategy. Notwithstanding, one of the main criticisms raised to this study is about the strategy adopted to achieve PWI. Due to the different thicknesses and complex anatomical orientations of the myocardial fibers within the PW, creating a standard linear lesion set may not be enough. Indeed, previous studies have reported a high reconnection rate when PWI is carried out using a “box” lesion set and low power (20–35 W). In contrast to CAPLA, the PRECEPT study reported a single-procedure success rate in Pe-AF of 80.4% at 15 months, with subsequent improvement in quality of life and a reduction in hospitalization, likely due to different ablation techniques [34,35]. Winkle et al. reported a large real-world evaluation of the combination of HPSD and CF sensing catheters [36]. They reported 1- and 4-year freedom-from-AF after initial ablation of 74.2% and 63.2% and after the final ablation of 82.1% and 71.9% for Pe-AF.

## 5. Limitations of the Study

This study has several limitations. First, this was a prospective non-randomized single-center study, and the number of patients included is limited. The study proves the feasibility of HPSD LSI-guided PWI beyond PVI. Although we compared the treatment group with a historical control group, we cannot give any definitive conclusion on the role of HPSD in performing PWI beyond PVI. Larger and randomized data and longer follow-up durations are needed to validate these data. Finally, a significant number of patients were on continuous AAD treatment even after the blanking period, preventing us from properly assessing the correlation between PWI and outcome.

## 6. Conclusions

LSI is an effective marker of lesion quality, giving more detailed information regarding lesion quality than CF only. PVI plus PWI using HPSD settings and guided by LSI performed during an index ablation of Pe-AF seems safe, effective, and reproducible. Our findings need to be validated in larger and randomized studies to confirm if this strategy should be adopted when performing the ablation of Pe-AF.

## Figures and Tables

**Table 1:** Baseline clinical characteristics.

	Overall Population (*n* = 35)	Historical Control Group (*n* = 46)
Male, *n* (%)	28 (80)	33 (71.7)
Age, mean ± SD	58.4 ± 11.6	61.2 ± 10.9
Duration of AF, months (mean ± SD)	11.6 ± 3.4	10.1 ± 4.1
Hypertension, *n* (%)	22 (62.8)	27 (58.7)
Diabetes, *n* (%)	4 (11.4)	5 (10.8)
Renal failure, *n* (%)	1 (2.8)	2 (4.3)
Dyslipidemia, *n* (%)	12 (34.2)	15 (32.6)
OSAS, *n* (%)	7 (20)	8 (17.4)
COPD, *n* (%)	3 (8.5)	3 (6.5)
Active smoker, *n* (%)	8 (22.8)	6 (13)
BMI, mean ± SD	29.6 ± 5.4	28.9 ± 4.8
CHA_2_DS_2_-VASc, mean ± SD	3.3 ± 0.9	3.1 ± 1.1
HASBLEED score, mean ± SD	1.6 ± 0.5	1.8 ± 0.8
LA diameter, mm (mean ± SD)	47.5 ± 13.2	48.2 ± 14.1
LA area, cm^2^ (mean ± SD)	31.8 ± 9.2	32.1 ± 8.8
LA volume, mL (mean ± SD)	66.8 ± 14.8	64.6 ± 15.1
Indexed LA volume, mL/m^2^ (mean ± SD)	32.8 ± 7.4	33.1 ± 6.9
LVEF, mean ± SD	56.8 ± 12.3	58.2 ± 10.1
Tachycardiomyopathy, *n* (%)	4 (11.4)	3 (6.5)
EHRA class IIa, *n* (%)	6 (17.2)	8 (17.4)
EHRA class IIb, *n* (%)	21 (60)	29 (63)
EHRA class III, *n* (%)	8 (22.8)	9 (19.5)
ICM, *n* (%)	5 (14.2)	6 (13)
DCM, *n* (%)	2 (5.7)	4 (8.7)
HCM, *n* (%)	1 (2.8)	0
*Baseline therapy*		
- Beta-blockers, *n* (%)	14 (40)	18 (39.1)
- Class IC, *n* (%)	3 (8.5)	5 (10.8)
- Amiodarone, *n* (%)	26 (74.3)	34 (73.9)
- Sotalol, *n* (%)	5 (14.2)	7 (15.2)

AF = atrial fibrillation; OSAS = obstructive sleep apnea syndrome; COPD = chronic obstructive pulmonary disease; BMI = body mass index; LA = left atrium; LVEF = left ventricular ejection fraction; ICM = ischemic cardiomyopathy; HCM = hypertrophic cardiomyopathy; PM = pacemaker.

**Table 2:** Procedural characteristics.

	Overall Population (*n* = 35)
Pre-procedural TEE, *n* (%)	35 (100)
Procedural duration, min (mean ± SD)	116.2 ± 10.9
Total RF time, min (mean ± SD)	22.8 ± 3.6
ICE, *n* (%)	5 (14.2)
US-guided femoral puncture, *n* (%)	8 (22.8)
Double transeptal puncture, *n* (%)	33 (94.2)
***PVI***	
LPV common ostia, *n* (%)	3 (8.5)
RPV common ostia, *n* (%)	0
Intermediate/accessory PVs, *n* (%)	1 (2.8)
PVI, *n* (%)	35 (100)
WACA, *n* (%)	4 (11.4)
WACA + carina, *n* (%)	31 (88.5)
PVs isolated at first-pass during PVI, *n* of PVs (%)	126/135 (93.3)
CF on anterior LPVs, (mean ± SD)	12.9 ± 3.4
CF on posterior LPVs, (mean ± SD)	10.8 ± 2.8
LSI on anterior LPVs, (mean ± SD)	5.2 ± 0.3
LSI on posterior LPVs, (mean ± SD)	4.4 ± 0.3
CF on anterior RPVs, (mean ± SD)	14.7 ± 2.4
CF on posterior RPVs, (mean ± SD)	11.3 ± 2.6
LSI on anterior RPVs, (mean ± SD)	5.3 ± 0.4
LSI on posterior RPVs, (mean ± SD)	4.6 ± 0.3
Adenosine, *n* (%)	35 (100)
PV acute reconnection, *n* (%)	2 (5.7)
***PWI***	
PWI, *n* (%)	35 (100)
RF time on PW, (mean ± SD)	5.3 ± 1.5
First-pass roofline block, *n* (%)	31 (88.5)
First-pass bottom line block, *n* (%)	18 (51.4)
First-pass PWI, *n* (%)	13 (37.1)
CF on PW, g (mean ± SD)	10.9 ± 2.6
LSI on PW, mean ± SD	4.4 ± 0.3
Adenosine, *n* (%)	35 (100)
PW acute reconnection, *n* (%)	3 (8.5)

TEE = transesophageal echocardiography; ICE = intracardiac echocardiography; US = ultrasound; LPV = left pulmonary vein; RPV = right pulmonary vein; PVI = pulmonary vein isolation; WACA = wide antral circumferential ablation; PW = posterior wall; LA = left atrium; LVEF = left ventricular ejection fraction, CF = contact force; LSI = lesion size index.

**Table 3:** HPSD vs. LPLD (control group).

	HPSD (*n* = 35)	LPLD (*n* = 46)	*p*
Procedural duration, min (mean ± SD)	116.2 ± 10.9	144.5 ± 11.3	<0.001
Total RF time, min (mean ± SD)	19.8 ± 3.6	26.3 ± 6.4	<0.001
RF time on PVs, min (mean ± SD)	14.2 ± 2.6	19.6 ± 3.2	<0.05
RF time on PW, min (mean ± SD)	5.3 ± 1.5	6.9 ± 1.6	<0.05
Fluoroscopy time, min (mean ± SD)	4.2 ± 3.1	4.6 ± 2.9	*ns*
Double transeptal puncture, *n* (%)	33 (94.2)	40 86.9)	*ns*
PVs isolated at first-pass during PVI, % (*n* of PVs)	93.3 (126/135)	94.9 (169/178)	*ns*
PV acute reconnection, *n* (%)	2 (5.7)	4 (8.7)	*ns*
First-pass roofline block, *n* (%)	31 (88.5)	41 (89.1)	*ns*
First-pass bottom line block, *n* (%)	18 (51.4)	21 (45.6)	*ns*
First-pass PWI, *n* (%)	13 (37.1)	17 (36.9)	*ns*
PW acute reconnection, *n* (%)	3 (8.5)	6 (13)	*ns*

RF = radiofrequency; PVs = pulmonary veins; PW = posterior wall; PVI = pulmonary vein isolation; PWI = posterior wall isolation; ns = non significant

## Data Availability

Data are available upon reasonable request.
